# Supplementary material for: Subsarcomeric regulation of thin and thick filaments in skeletal muscle myofibrils
Source: Proc Natl Acad Sci U S A. 2026 Jun 2;123(23):e2535527123. doi: 10.1073/pnas.2535527123 (PMC13250608; doi:10.1073/pnas.2535527123)
Supplement: Supplementary file 1 — Appendix 01 (PDF) [file pnas.2535527123.sapp.pdf]

PNAS

Supporting Information for

Subsarcomeric regulation of thin and thick filaments in skeletal muscle myofibrils.

Kristina Sliogeryte, Luca Fusi

*Corresponding author: Luca Fusi.
Email: luca.fusi@kcl.ac.uk

This PDF file includes:

Supporting text
Figures S1 to S8
Tables S1 to S3
SI References

Supporting Information Text

Simulation of the spatial distributions of RLC and TnC orientations along thick and thin filaments, respectively, in skeletal myofibrils.

To calculate the spatial distribution of orientation of RLC and TnC probes along thick and thin filaments, respectively, we assumed a Gaussian distribution for the fluorescence intensity of single fluorophores along the filament:

$$I(x) = e^{\frac{-(x-x_0)^2}{2\sigma^2}}$$

in which σ is determined by the point spread function of our optical system and x_0 is the position of each probe along the filament. Using fluorescent beads with diameter=170 nm as point sources (P7220, Invitrogen) we determined σ=200 nm (FWHM=470 nm) for the point spread function of our microscope (Fig. S2), which is consistent with the theoretical value for the spatial resolution (SR) of about 430 nm with NA=0.7 and wavelength of fluorescence (λ=620 nm) (SR=λ/2NA) .

To calculate the distribution of $<P_2>$ values across the Z-band and the M-band for TnC and RLC probes, respectively, we assumed homogenous labelling (Model 1; Fig. S1) of troponin and myosin along thin and thick filaments, respectively. Specifically, each thin filament contained 30 TnC fluorophores, beginning 70 nm from the centre of the Z-band with a spacing of 38 nm, while each half thick filament contained 49 RLC fluorophores, starting 80 nm from the M-line with a spacing of 14.5 nm (Fig. S2). To account for contributions from adjacent sarcomeres, additional TnC and RLC arrays were placed at distances of ±SL, where SL is the sarcomere length measured experimentally in the myofibril.
The spatial distribution of $<P_2>$ for the RLC probe in each half thick filament was calculated as:

$$P_{2\_RLC}(x) = \frac{\sum_0^{11}(< P_2 >_P \cdot I_i) + \sum_{12}^{29}(< P_2 >_C \cdot I_i) + \sum_{30}^{48}(< P_2 >_D \cdot I_i)}{\sum_0^{48} I_i + I_{iso}}$$

in which I_i is the fluorescence intensity distribution for the RLC probe at each myosin layer (i), I_{iso} is a constant fluorescence intensity distribution along the sarcomere produced by a population of isotropic ($<P_2>$=0) and non-specifically bound probes, $<P_2>_P$, $<P_2>_C$ and $<P_2>_D$ are the $<P_2>$ values for probes in the P (myosin layers 0-11), C (layers 12-29) and D (layers 30-48) zones of the filament (Fig. S2). I_{iso} was fixed to ~10% of the total maximal fluorescence intensity produced by specifically bound probes. Therefore $<P_2>_P$, $<P_2>_C$ and $<P_2>_D$ are the only free parameters that were adjusted to fit the experimental spatial distributions of RLC orientations.
Similarly, the orientation distributions of $<P_2>$ for the TnC probes in rigor and relaxed myofibrils shown in Fig.1 were calculated as:

$$P_{2\_TnC}(x) = \frac{\sum_0^{11}(< P_2 >_I \cdot I_i) + \sum_{12}^{29}(< P_2 >_A \cdot I_i)}{\sum_0^{29} I_i + I_{iso}}$$

in which $<P_2>_I$, $<P_2>_A$ are $<P_2>$ values for TnC probes in the I- (troponin 0-11) and A-bands (troponin 12-29) of the sarcomere (Fig. S2).

The best fit to the $<P_2>$ distribution was determined by a search of the zonal $<P_2>$ values by minimizing chi-square (χ^2), calculated as:

$$\chi 2 = \sum \frac{(O-E)^2}{E}$$

where *O* and *E* are observed and simulated values of <P_2>, respectively.

The normalized fluorescence intensity distribution along the filament was calculated as:

$$I_{tot}(x) = \frac{\sum_0^i I_i + I_{iso}}{I_{max}}$$

A better fit to the spatial distribution of fluorescence intensity for the RLC and TnC probes was obtained by assuming a non-uniform distribution of probes along the filament (Model 2; Fig. S1), due to differences in the efficiency of RLC and TnC exchange across filament domains (see methods). Specifically, the RLC intensity distribution was reproduced by assuming that the fluorescence intensity at each myosin layer in the P-zone was 2.5-fold higher than in the C- and D-zones. For the TnC probes, the fluorescence intensity in each troponin layer within the I-band was assumed to be 1.5-fold and 1.2-fold higher than in the A-band for TnC-C and TnC-E, respectively. However, the <P_2> distribution for each probe was largely insensitive to the distribution of probes along the filaments (Fig. S1).

Figures

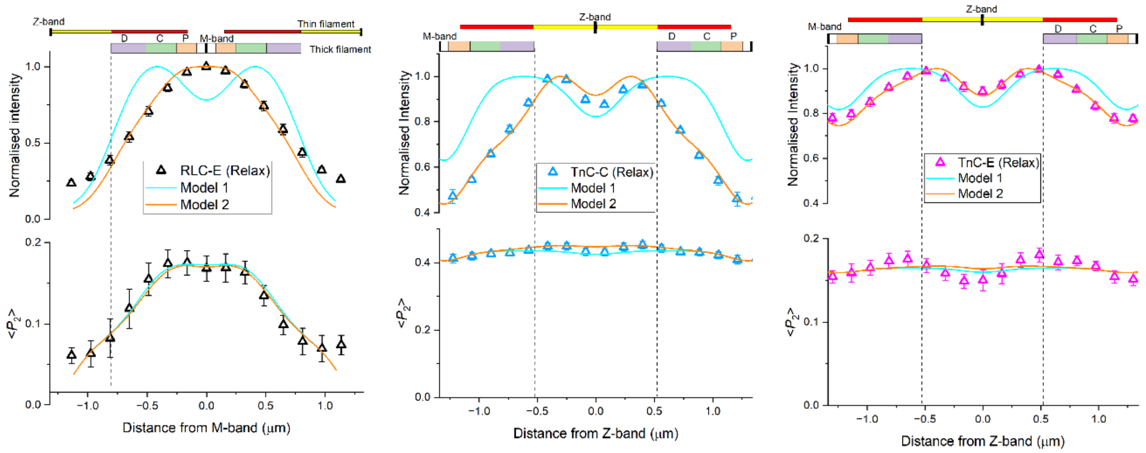

Fig. S1. Distribution of Intensity and <P_2> for RLC-E probe (black triangles) along the thick filament and for TnC-C (cyan triangles) and TnC-E (magenta triangles) probes along the thin filaments in the sarcomere of relaxed (pCa 9) myofibrils at 17°C. Cyan solid lines represent the simulated intensity and <P_2> for each probe using the model described in the Supporting Information Text, assuming a uniform probe distribution along thin and thick filaments (Model 1). Orange solid lines represent the simulated intensity and <P_2> for each probe, assuming a non-uniform distribution along the filaments (Model 2): for RLC, the number of probes in each myosin layer of the P-zone was assumed to be 2.5-fold higher than in the C- and D-zones; for TnC probes, the number of probes in each troponin layer within the I-band was assumed to be 1.5-fold and 1.2-fold higher than in the A-band for TnC-C and TnC-E, respectively. Note that, although the intensity distribution of each probe is better reproduced by Model 2, the distribution of <P_2> for each probe is largely insensitive to the distribution of probes along the filaments.

A

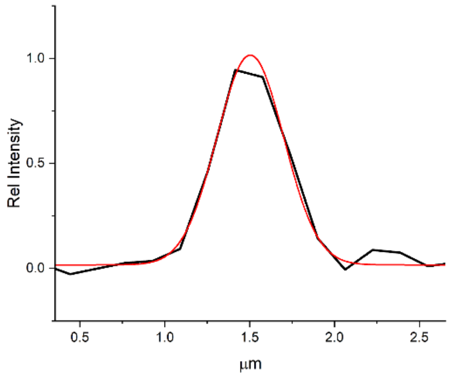

B

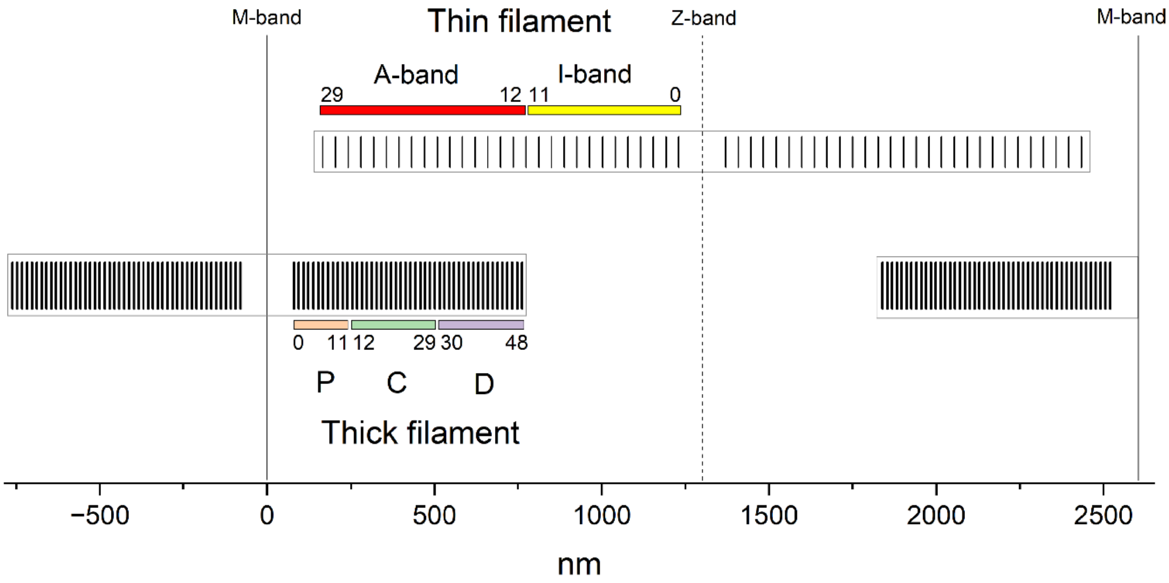

Fig. S2. A) Microscope point spread function measured by Gaussian fit (red) of the fluorescence intensity distribution (black) of a point source (fluorescent bead, 170nm diameter). B) TnC and RLC probe arrays on thin and thick filaments, respectively, used for the calculation of the spatial distribution of probe orientation in myofibrils (see Supplementary Text). Each probe position on the filaments is marked by small vertical lines. Colored squares mark P, C, D zones of the thick filaments and A- and I-band domains of the thin filament, with indexes of probe positions at the boundaries of each zone. The sarcomere length measured in the myofibril in each condition was used to set the distance between arrays in adjacent sarcomeres. In the schematic presented above the sarcomere length is 2.6 µm.

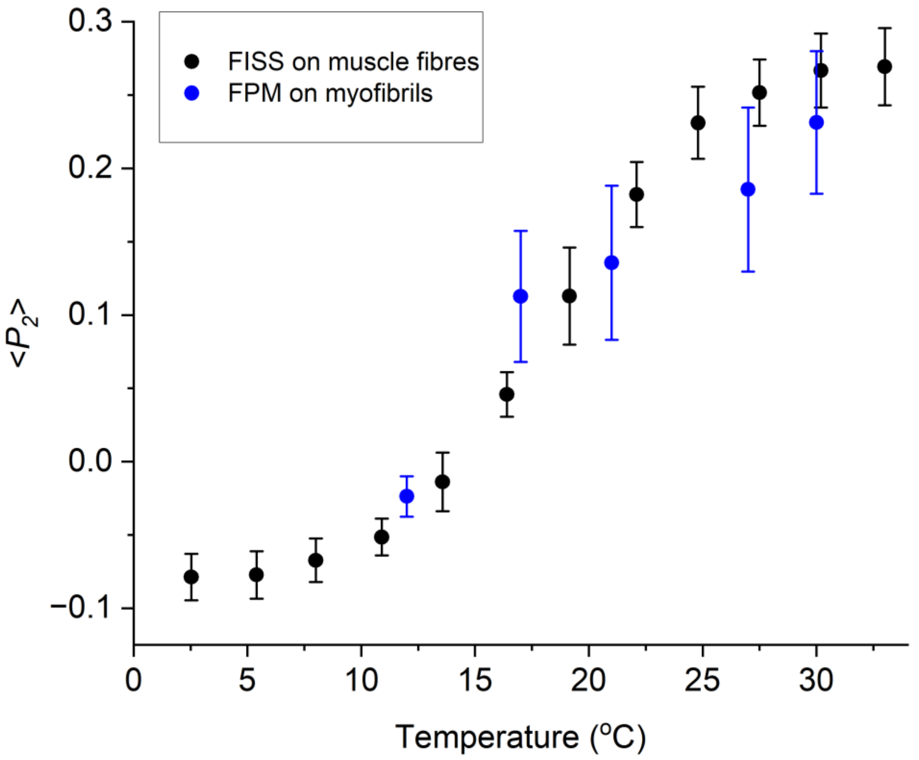

Fig. S3. Comparison of the temperature dependence of bulk <P_2> values for the RLC-E probe measured by FISS in demembranated muscle fibers from rabbit psoas (From Fusi et al., 2015)[1] and space-averaged <P_2> measured by FPM in a single sarcomere of skeletal myofibrils from rabbit psoas (Fig.2A) under relaxing conditions (pCa 9) in the absence of Dextran.

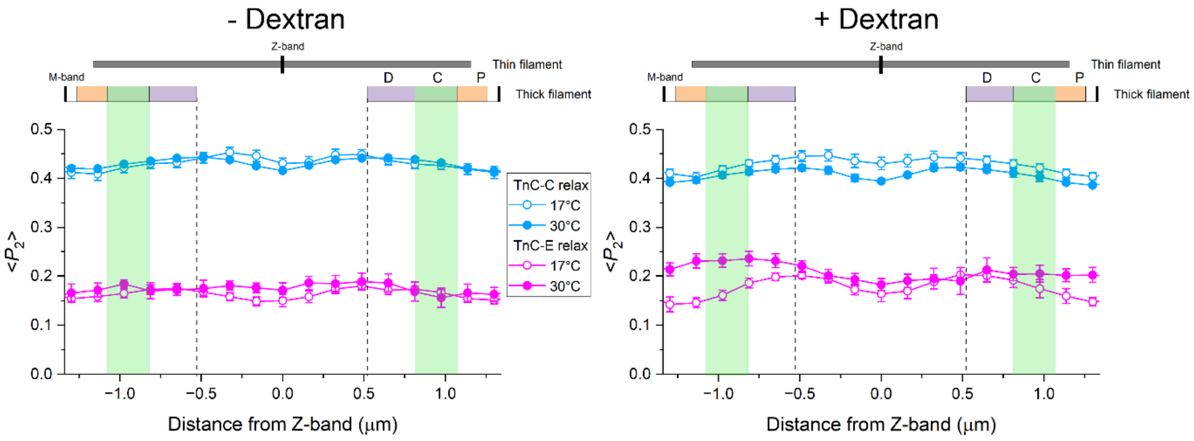

Fig. S4. Average distribution of <P_2> (mean±SE) for TnC-C (cyan) and TnC-E (magenta) probes along the thin filaments in the relaxed sarcomere (pCa 9.0) of single myofibrils at 17°C (n=5 myofibrils for TnC-C and n=6 for TnC-E) and 30°C (n=5 for TnC-C and n=7 for TnC-E), in the absence and presence of 4% (w/v) Dextran T-500; sarcomere length= 2.52±0.04 μm.

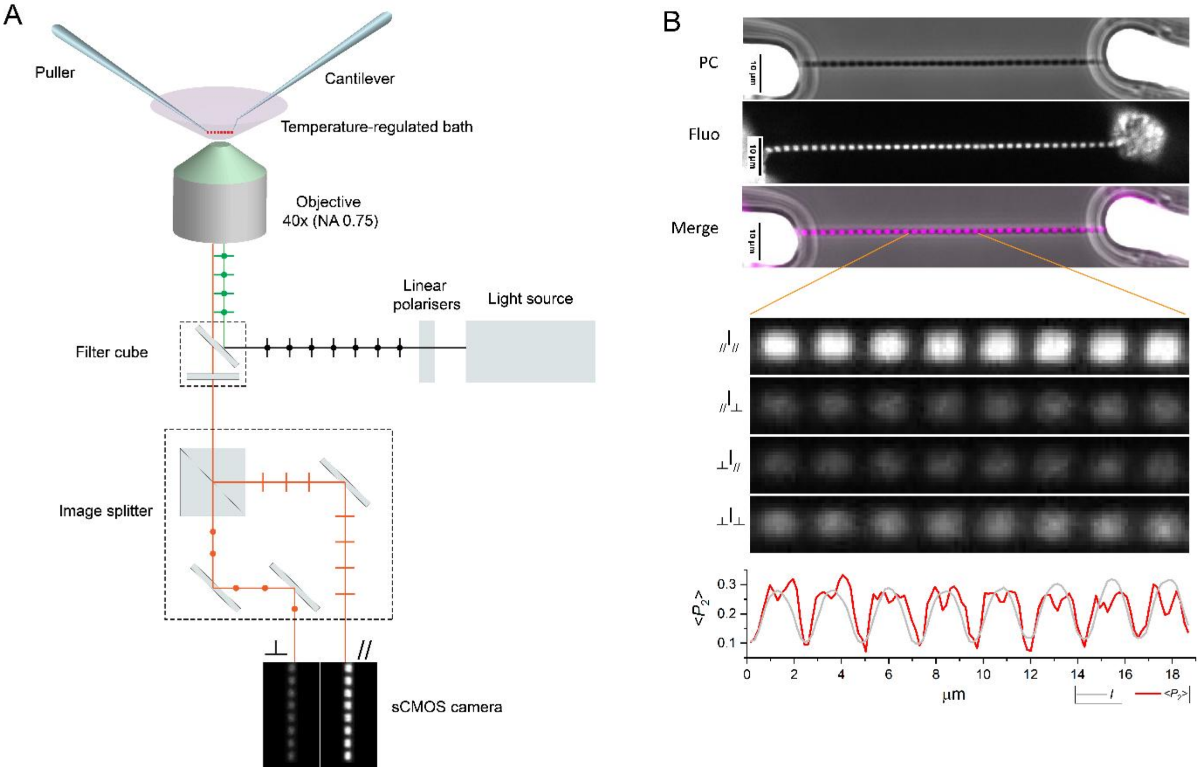

Fig. S5. A) Schematic of the Fluorescence Polarization Microscope and myofibril setup. B) Phase contrast (PC) and fluorescence (Fluo) images of a skeletal myofibril from rabbit psoas exchanged with the RLC-E probe in relaxing solution at 30°C. Lower panels, polarized images of sarcomeres in the myofibril (100ms exposure) and the calculated 1D-spatial distribution of $<P_2>$ and total fluorescence intensity (I) along the myofibril axis (see methods).

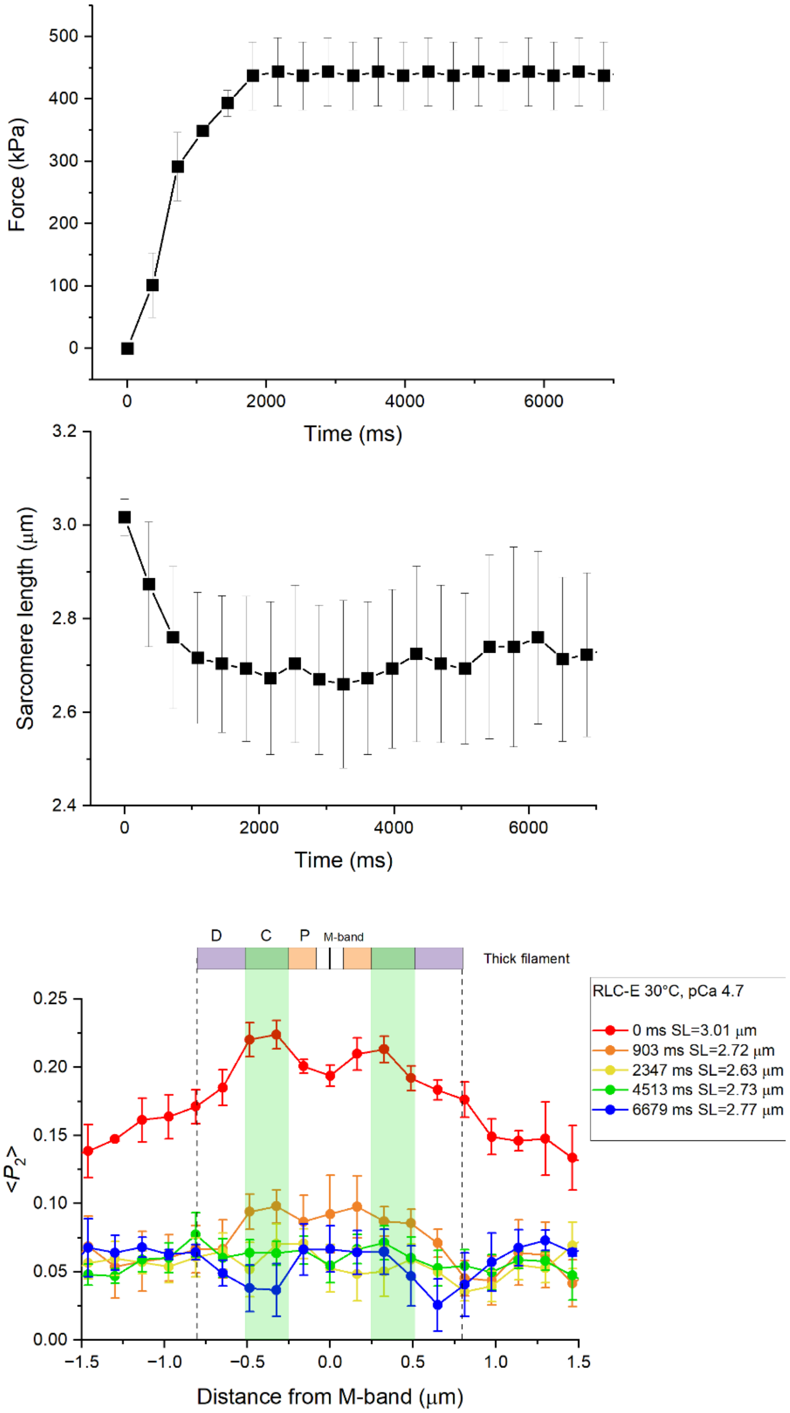

Fig. S6. Time courses of force, sarcomere length and spatial distributions of <P_2> (mean±SE) for the RLC-E probe along the thick filament, in myofibrils activated at pCa 4.7 at 30°C (n=3 myofibrils).

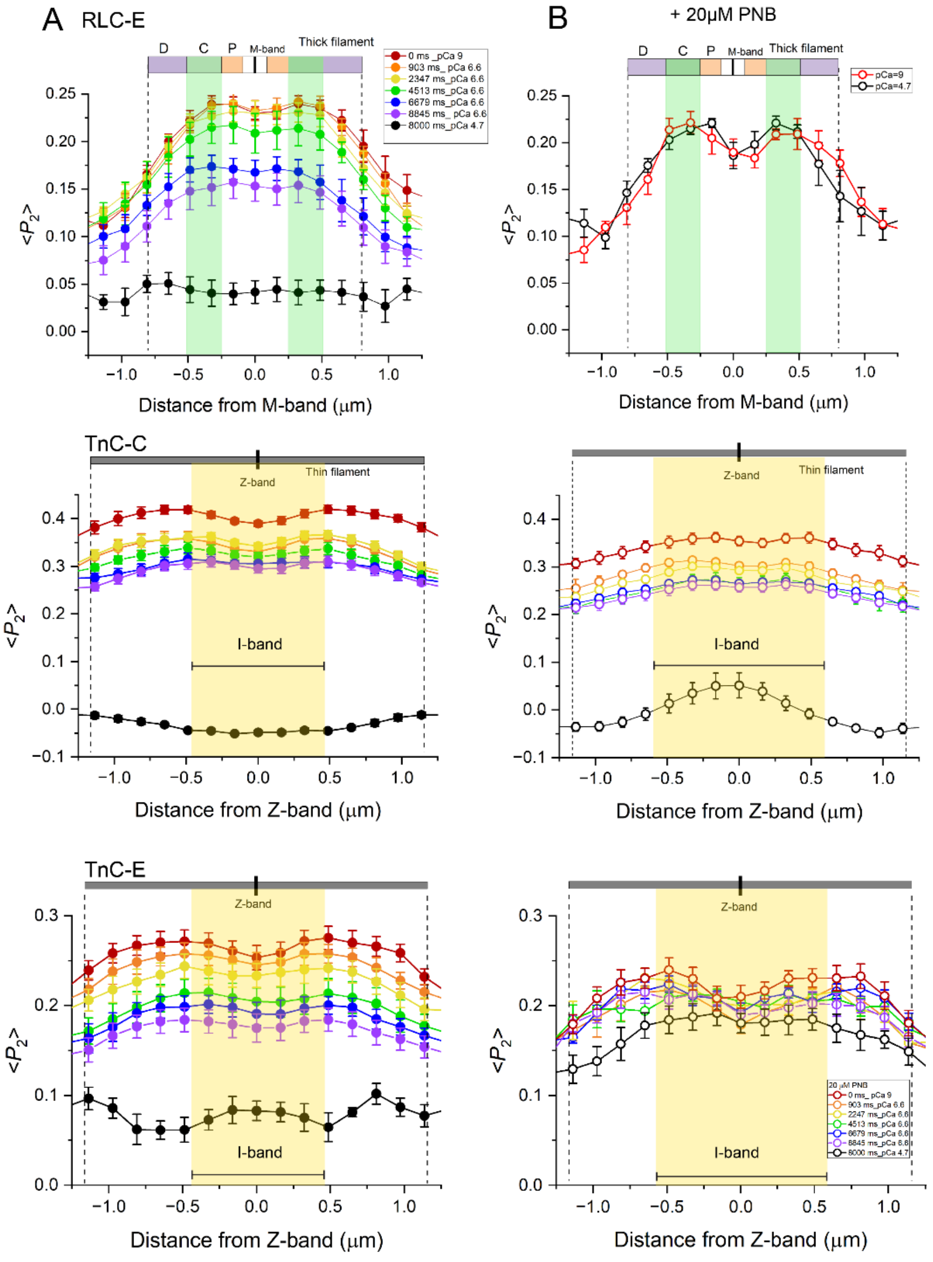

Fig. S7. Time courses of the changes in the spatial distributions of <P_2> (mean±SE) for RLC and TnC probes along thick and thin filaments, respectively, in myofibril activated at pCa 4.7 and 6.6 at 30°C (Fig. 4) in the absence (left, A) and presence (right, B) of 20 μM Para-Nitro Blebbistatin (PNB). Green squares, thick filament C-zone. Yellow squares, I-band width.

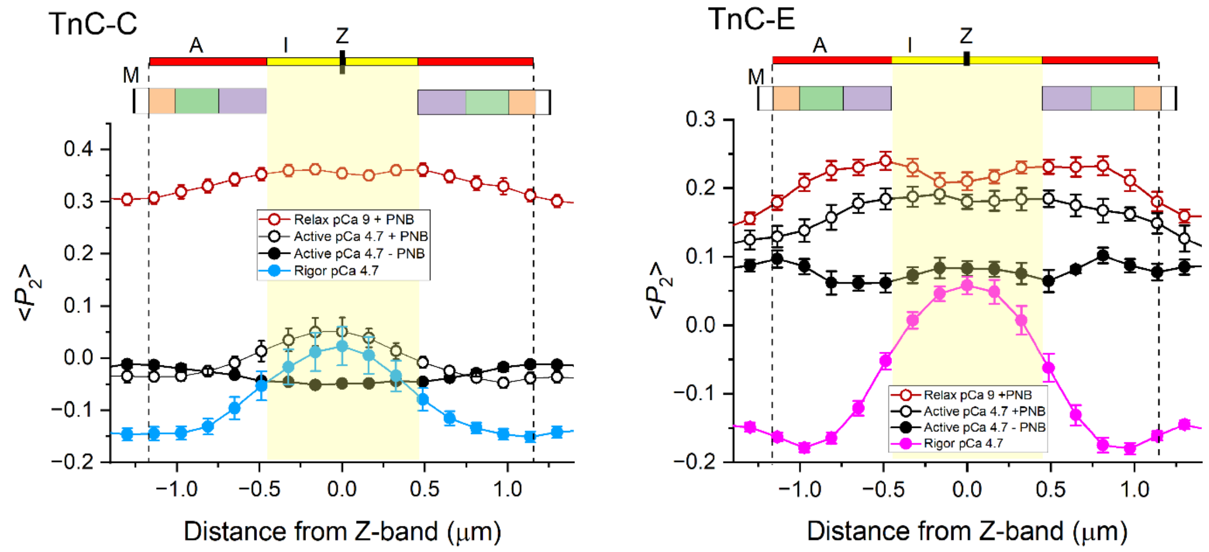

Fig. S8. Comparison of the orientation distribution of TnC-C and TnC-E probes along thin filaments in I- (yellow) and A- (red) bands in the sarcomere of relaxed (pCa 9), and active (pCa 4.7) myofibrils in the presence of PNB (data from Fig.5), in active myofibrils (pCa 4.7) in the absence of PNB (data from Fig. 4) and in rigor myofibrils at pCa 4.7 (cyan, data from Fig.1).

Tables

	$<P_2>$	
**TnC-C**	**FISS (muscle fibres)**	**FPM (myofibrils)**
Relax	0.422 ± 0.021[†]	0.431 ± 0.012
Rigor pCa 9.0	0.207 ± 0.018[†]	0.232 ± 0.009
Rigor pCa 4.7	-0.142 ±0.007[†]	-0.088 ± 0.022
Active pCa 4.7	-0.133 ± 0.004[§]	-0.032 ± 0.005

**TnC-E**	**FISS (muscle fibres)**	**FPM (myofibrils)**
Relax	0.196 ± 0.007[†]	0.210 ± 0.010
Rigor pCa 9.0	-0.005 ± 0.012[†]	-0.023 ± 0.007
Rigor pCa 4.7	-0.123 ± 0.014[†]	-0.089 ± 0.010
Active pCa 4.7	-0.017 ± 0.009[§]	0.078 ± 0.010

**RLC-E**	**FISS (muscle fibres)**	**FPM (myofibrils)**
Relax	0.113± 0.033*	0.113 ± 0.016
Rigor pCa 9.0	-0.290 ± 0.024*	-0.220 ± 0.004
Active pCa 4.7	0.020 ± 0.012[§]	0.040 ± 0.011

Table S1. Comparison of published bulk $<P_2>$ values for TnC and RLC probes measured by wide-field Fluorescence for In-Situ Structure (FISS) in rabbit psoas muscle fibers with corresponding space-averaged $<P_2>$ values measured by Fluorescence Polarization Microscopy (FPM) in single sarcomeres of skeletal muscle myofibrils from rabbit psoas. Relaxed and rigor data are at 17°C; active data are at 27-30°C.[†]TnC FISS data are from Sun et al. 2006[2]; *RLC FISS data are from Fusi et al. 2015[1]; [§] RLC/TNC FISS data from Brunello et al. 2023[3].

TnC-C	$\langle P_2 \rangle$ I-band	$\langle P_2 \rangle$ A-band
Relax	0.506 ± 0.015	0.506 ± 0.015
Rigor pCa 9.0	0.415 ± 0.009*	0.171 ± 0.007*,§
Rigor pCa 4.7	0.029 ± 0.050*,†	-0.179 ± 0.015*,§,†

TnC-E	$\langle P_2 \rangle$ I-band	$\langle P_2 \rangle$ A-band
Relax	0.193 ± 0.009	0.198 ± 0.009
Rigor pCa 9.0	0.098 ± 0.011*	-0.099 ± 0.006*,§
Rigor pCa 4.7	0.074 ± 0.014*	-0.206 ± 0.008*,§,†

RLC-E	$\langle P_2 \rangle$_P	$\langle P_2 \rangle$_C	$\langle P_2 \rangle$_D
Relax	0.193 ± 0.019^{e}	0.200 ± 0.023$^{\$}$	0.083 ± 0.020
Rigor pCa 9.0	-0.194 ± 0.012$^{*,\circ,\bullet}$	-0.298 ± 0.008*	-0.270 ± 0.004*
Rigor pCa 4.7	-0.190 ± 0.017$^{*,\circ,\bullet}$	-0.286 ± 0.006*	-0.273 ± 0.005*

Table S2. $\langle P_2 \rangle$ values (mean ± SE) for TnC probes in A/I bands and RLC probes in P/C/D zones at 17°C derived from model simulations of the orientation distributions in Fig. 1. Superscripted symbols denote significant differences in $\langle P_2 \rangle$ using ANOVA with Tukey's post hoc analysis. *$P<0.05$ when comparing $\langle P_2 \rangle$ in rigor and relaxing conditions. $^{\S}P<0.05$ when comparing $\langle P_2 \rangle$ for TnC probes in I- and A-bands. $^{\dagger}P<0.05$ when comparing $\langle P_2 \rangle$ for TnC probes in rigor pCa 9 and pCa 4.7. $^{\$}P<0.05$ when comparing $\langle P_2 \rangle$ for RLC-E probe in C- and D-zones. °$P<0.05$ when comparing $\langle P_2 \rangle$ for RLC-E probe in P- and D-zones. $^{\bullet}P<0.05$ when comparing $\langle P_2 \rangle$ for RLC-E probe in P- and C-zones.

	-PNB		+PNB	
**TnC-C**	$<P_2>$ I-band	$<P_2>$ A-band	$<P_2>$ I-band	$<P_2>$ A-band
pCa 9.0	0.478 ± 0.010	0.478 ± 0.010	0.422 ± 0.010 *	0.398 ± 0.017 *
pCa 6.6	0.355 ± 0.013	0.340 ± 0.014	0.296 ± 0.016 *	0.268 ± 0.011 *
pCa 4.7	-0.061 ± 0.005	-0.019 ± 0.011	0.062 ± 0.035 $^{*,\$}$	-0.051 ± 0.012

	-PNB		+PNB	
**TnC-E**	$<P_2>$ I-band	$<P_2>$ A-band	$<P_2>$ I-band	$<P_2>$ A-band
pCa 9.0	0.311 ± 0.017	0.311 ± 0.017	0.255 ± 0.013	0.263 ± 0.015
pCa 6.6	0.208 ± 0.018	0.206 ± 0.017	0.236 ± 0.010	0.236 ± 0.010
pCa 4.7	0.095 ± 0.015	0.080 ± 0.010	0.208 ± 0.019 *	0.200 ± 0.017 *

	-PNB			+PNB		
**RLC-E**	$<P_2>$_P	$<P_2>$_C	$<P_2>$_D	$<P_2>$_P	$<P_2>$_C	$<P_2>$_D
pCa 9.0	0.222 ± 0.004	0.288 ± 0.017 §,†	0.182 ± 0.012	0.203 ± 0.011	0.273 ± 0.011 §,†	0.203 ± 0.011
pCa 6.6	0.154 ± 0.017	0.178± 0.022 §	0.106 ± 0.021	-	-	-
pCa 4.7	0.046 ± 0.012	0.049 ± 0.011	0.039 ± 0.011	0.185 ± 0.010 ^{a}	0.280 ± 0.004 *,§,†	0.175 ± 0.005 *

Table S3. $<P_2>$ values (mean ± SE) for TnC probes in A/I bands and RLC probes in P/C/D zones at 30°C derived from model simulations of the orientation distributions in the absence (Fig. 4) and in the presence of 20μM PNB (Fig. 5). Superscripted symbols denote significant differences in $<P_2>$ using ANOVA with Tukey's post hoc analysis. *P<0.05 when comparing $<P_2>$ in a filament domain in the absence and presence of PNB. §P<0.05 when comparing $<P_2>$ for RLC-E probe in C- and D-zones. †P<0.05 when comparing $<P_2>$ for RLC-E probe in C- and P-zones. $^{\$}$P<0.05 when comparing $<P_2>$ for TnC probes in I- and A-band.

SI References

1. L. Fusi, Z. Huang, M. Irving, The Conformation of Myosin Heads in Relaxed Skeletal Muscle: Implications for Myosin-Based Regulation. *Biophys. J.* **109**, 783-792 (2015).
2. Y. B. Sun, B. Brandmeier, M. Irving, Structural changes in troponin in response to Ca2+ and myosin binding to thin filaments during activation of skeletal muscle. *Proc Natl Acad Sci USA* **103**, 17771-17776 (2006).
3. E. Brunello, L. Marcucci, M. Irving, L. Fusi, Activation of skeletal muscle is controlled by a dual-filament mechano-sensing mechanism. *PNAS* **120**, e2302837120 (2023).
